# Weak Interaction of the Antimetabolite Drug Methotrexate with a Cavitand Derivative

**DOI:** 10.3390/ijms21124345

**Published:** 2020-06-18

**Authors:** Zsolt Preisz, Zoltán Nagymihály, Beáta Lemli, László Kollár, Sándor Kunsági-Máté

**Affiliations:** 1 Institute of Organic and Medicinal Chemistry, Medical School, University of Pécs, Szigeti 12, H-7624 Pécs, Hungary; preisz.zsolt@pte.hu (Z.P.); beata.lemli@aok.pte.hu (B.L.); 2 Department of General and Physical Chemistry, Faculty of Sciences, University of Pécs, Ifjúság 6, H 7624 Pécs, Hungary; 3 Department of Inorganic Chemistry, Faculty of Sciences, University of Pécs, Ifjúság 6, H 7624 Pécs, Hungary; nmzoltan@gamma.ttk.pte.hu (Z.N.); kollar@gamma.ttk.pte.hu (L.K.); 4 János Szentágothai Research Center, University of Pécs, Ifjúság 20, H-7624 Pécs, Hungary

**Keywords:** methotrexate, antirheumatic, cavitand, inclusion complex, thermodynamics, fluorescence

## Abstract

Formation of inclusion complexes involving a cavitand derivative (as host) and an antimetabolite drug, methotrexate (as guest) was investigated by photoluminescence measurements in dimethyl sulfoxide solvent. Molecular modeling performed in gas phase reflects that, due to the structural reasons, the cavitand can include the methotrexate in two forms: either by its opened structure with free androsta-4-en-3-one-17α-ethinyl arms or by the closed form when all the androsta-4-en-3-one-17α-ethinyl arms play role in the complex formation. Experiments reflect enthalpy driven complex formation in higher temperature range while at lower temperature the complexes are stabilized by the entropy gain.

## 1. Introduction

**Figure 1:**
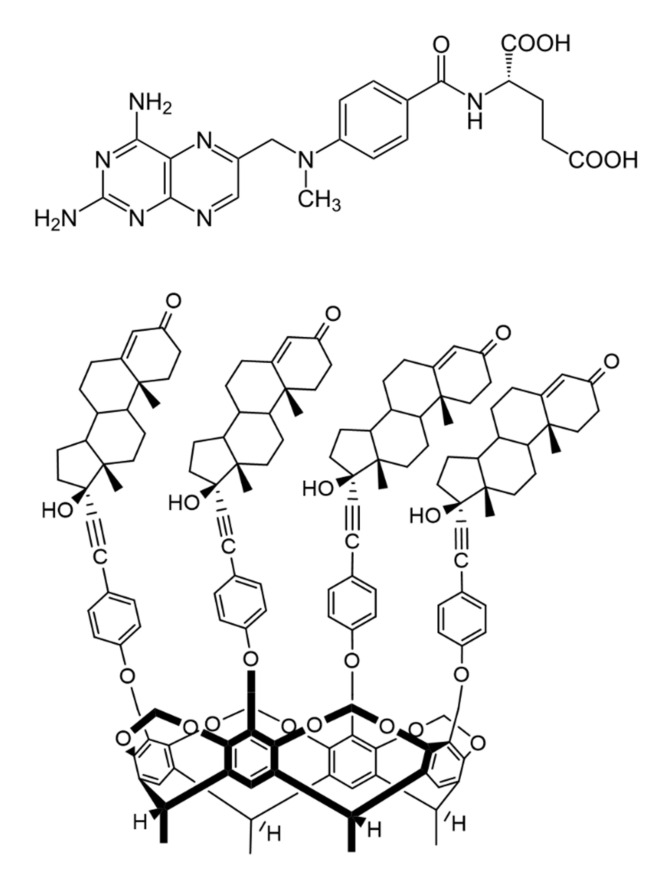
Chemical structure of antirheumatic and antitumor drug methotrexate (MTX, top) and the macrocyclic host molecule tetrakis(androst-4-en-3-one-17α-ethinyl)-cavitand (TAC, bottom) (8β-H, 9α-H and 13α-H are omitted for clarity).

Methotrexate (4-{*N*-[(2,4-diaminopteridin-6-yl) methyl]-*N*-methylamino} benzoyl)-*L*-glutamic acid, MTX, Figure 1) is an antimetabolite drug. It is widely used as a chemotherapeutic agent in rheumatoid arthritis (RA), psoriasis and some sorts of leukemia. MTX is a relatively well-known molecule and is a first-line antirheumatic medication because of its efficacy and safety [1]. It decreases the concentration of tetrahydrofolate (THF) in the cells by the inhibition of dihydrofolate reductase (DHFR) enzyme, therefore it reduces the purine nucleotide and DNA synthesis [2]. Other mechanisms of action were also mentioned in the literature, including reduction of antigen-dependent T-cell proliferation, promotion of adenosine release and suppression of transmethylation reaction [3,4].

Calixarenes are cyclic oligomers that consist of phenolic units that are condensated in the presence of an aldehyde and linked to each other, typically through a methylene bridge, in an acidic environment [5,6]. They are widely used in supramolecular chemistry, separation science and catalysis. They also have pharmaceutical applications as host molecules [6]. Substituents appended to the phenolic rings (upper or lower rim) can greatly influence the physical and chemical properties of these molecules, but numerous derivatives have been synthesized that have functional groups on the periphery of the molecule, too [7]. Promising results have been described in the literature about calixarenes that have heterocyclic substituents on the periphery, they can be used as selective extractants for amino acids [8], chiral recognition agents [9] or chelators that trap metals [10]. Calix[4] arene-based P-ligands were used in rhodium-catalyzed hydroformylation [11].

Inserting additional methylene bridges between the phenolic oxygen atoms on adjacent aromatic rings, the structure is called a cavitand.

There is a wide range of literature available on the interactions between MTX and some macrocyclic compounds, e.g., cyclodextrins [12,13,14] and cucurbiturils [15], but just a limited number of researches were reported about the calixarene derivatives [16]. Moreover, no publication was found to describe the interaction of MTX with cavitands, despite that calixarenes and cavitands have some advantages compared with other host molecules, because they possess an aromatic cavity, which can embed MTX due to the π–π and CH–π interactions both inside and outside the cavity [16].

The MTX-cavitand interaction found to be an entirely unrevealed research field. Therefore, in this work the thermodynamic parameters of the interaction of MTX with tetrakis(androst-4-en-3-one-17-α-ethinyl)-cavitand (TAC) (Figure 1) were studied. This is a calix[4] resorcinarene derivative cavitand, in which ethisterone moieties are appended to the aromatic rings. Fluorimetric measurements were applied to determine the thermodynamic parameters of the MTX-cavitand complex formation reaction.

## 2. Results

### 2.1. Spectroscopic Determination of the Association Constants

**Figure 2:**
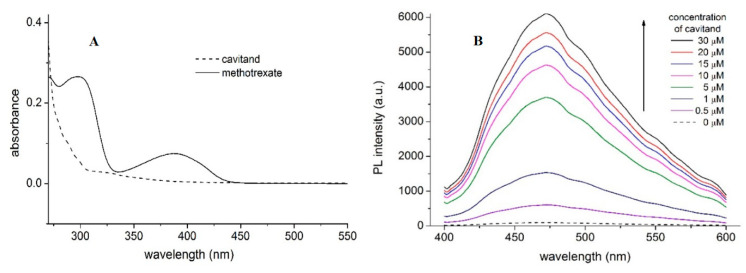
Absorption spectra of MTX (10 µM) and TAC (10 µM) (**A**) and fluorescence emission spectra of MTX (10 µM) in the absence and presence of TAC (0–30 µM) at 393.15 K (**B**) PL and a.u. reflect photoluminescence and arbitrary units, respectively.

**Figure 3:**
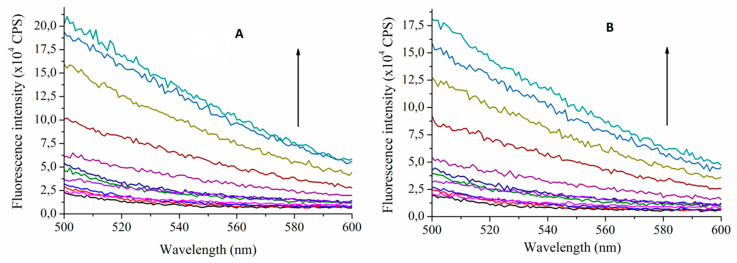
Fluorescence emission spectra of MTX (10 µM) in the absence and presence of TAC (0–80 µM) at 393.15 K (**A**) and at 313.15 K (**B**) (λ_excitation_ = 390 nm). Arrows indicate increasing TAC concentrations (CPS reflects counts per s, colors only support the clarity).

Absorption spectra of MTX and TAC (Figure 2A) show absorption maximum of MTX at 390 nm, while no considerable emission of TAC can be obtained at this wavelength. Accordingly, applying 390 nm excitation wavelength, emission spectra of MTX were recorded. Fluorescence spectra of MTX show increased emission upon increased concentration of TAC (Figure 3.). The enhancement of the PL signal can be described by two processes: (i) the guest MTX molecules loose the solvation shell reducing the quenching the PL signal induced by the solvent molecules, or, ii) the flexible MTX skeleton stabilized further by the TAC hosts and the reduced movements also support increasing the PL signal. Therefore, the changes induced in the spectra of MTX reflect interaction between the two molecules. 

The stability constants of this interaction can be calculated using the Benesi–Hildebrand method. To determine the thermodynamic parameters, the stability constants were calculated at temperatures 293.15 K, 296.48 K, 299.82 K, 303.15 K, 306.48 K, 309.82 K and 313.15 K. Considering that the DMSO solvent shows considerable Raman peaks overlapping the emission of MTX, two methods were applied to confirm the complex stabilities: either the HyperQuad code was applied using 10 selected wavelengths around the emission maxima (470–480 nm, see Figure 2.) or applying the Benesi–Hildebrand method, the right leg of the emission spectra is used, where the Raman scattering is negligible. In this case intensities obtained at 540 nm emission wavelength were applied to evaluate the data. Table 1 summarizes these results. These stability constants were then used to calculate the thermodynamic parameters of the interaction (Table 2).

### 2.2. Formation Thermodynamics

**Figure 4:**
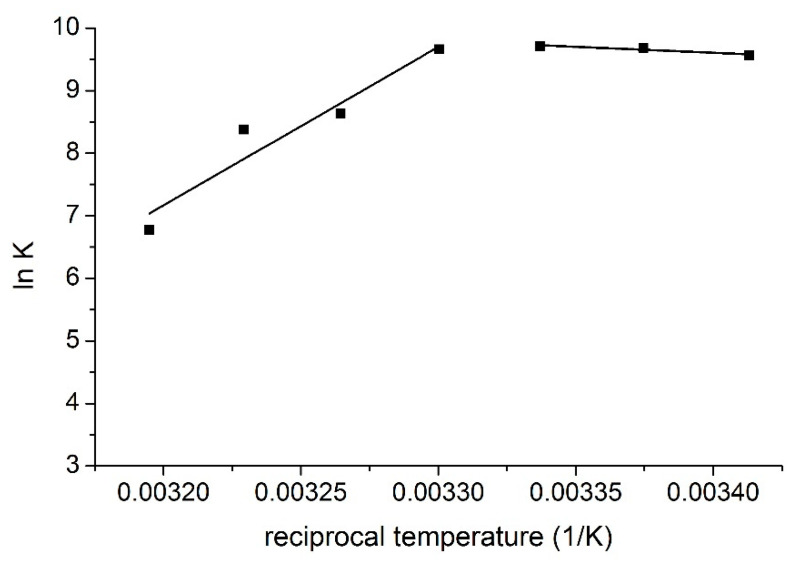
van ’t Hoff plot of the complex formation of MTX and TAC.

Two different temperature regions can be observed on the van ’t Hoff plot (the logarithms of the stability constants were plotted against the reciprocal temperatures, Figure 4) of the interaction where the thermodynamic parameters of the complex formation differ significantly.

These results imply different complex formation mechanisms in the different temperature regions. At lower temperatures, entropy gain is associated with enthalpy gain, but at higher temperatures, entropy loss is associated with enthalpy loss. Based on these observations, it is suggested that in the lower temperature region MTX interacts with the ethisterone moieties of TAC, i.e., with the chiral inlet of the host molecule. In this way, the entropy gain is caused by the removal of solvent molecules, but this process costs energy, this is the reason of the moderated positive enthalpy change. However, in the higher temperature region MTX interacts with the rigid inner cavity of TAC, i.e., with the lower lying aromatic ’basket’, which causes entropy loss associated with the decreased freedom of the cavitand skeleton during complex formation. The negative enthalpy change reflects attractive interactions between the MTX molecules and the ethisterone arms of the cavitand.

### 2.3. Modeling Studies

**Figure 5:**
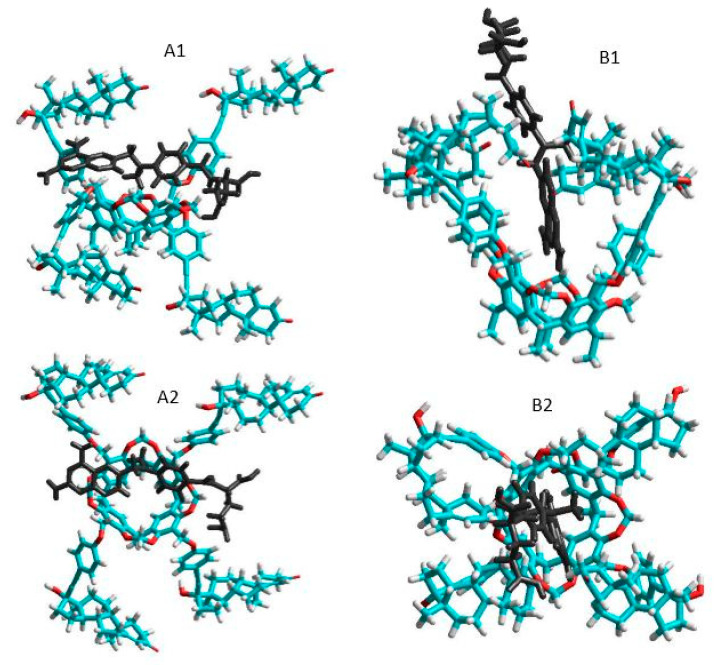
Side and top views of the equilibrium conformations of methotrexate–cavitand complexes associated with the opened (**A1** and **A2**) and closed (**B1** and **B2**) conformations of the cavitand (blue, red, white and black colors mean carbon, oxygen, hydrogen atoms and MTX molecule, respectively).

The total energies of the species interacted were calculated first at semi-empirical AM1 level using Hyper Chem 8 code. The host cavitand molecule shows two stable conformers according to the orientation of the large ethisterone moieties on the upper rim (see Figure 5): the opened structure associated with more flexible skeleton compared to the closed structure where the ethisterone moieties possess reduced freedom due to the steric hindering of their motion. The differences between the calculated total Gibbs free energies of the parent ’opened’ or ’closed’ molecules in gas phase (partition of solvent motions is excluded) was found to be 21.7 kJ/mole at 298.16 K, which property support presence of the opened structure exclusively at around room temperature.

The thermodynamic parameters associated with the complex formation of the TAC with MTX were then calculated as described in the Materials and methods section. Results show positive enthalpy and entropy change during formation of the closed form complexes, while both the enthalpy and entropy term is negative when the complex show opened structure. Possible reason for these results is that the freedom of the flexible opened cavitand decreased considerably when the closed form complexes are stabilized. In contrast, the entropy gain can be obtained when the flexibility of the cavitand skeleton remains unchanged during formation of the opened complex structure. The good agreement between the experimental results derived in solution phase and that of the theoretical value calculated in gas phase reflects moderated entropy gain associated with the increased freedom of solvent molecules after leaving the solvation shell of the methotrexate guest.

## 3. Materials and Methods

### 3.1. Chemicals

Methotrexate (MTX) was obtained from Sigma-Aldrich. Tetrakis (androst-4-en-3-one-17α-ethinyl)-cavitand (TAC) was synthesized in our institute according to a published method [17]. The applied solvent was dimethyl sulfoxide (DMSO) purchased from Merck (Darmstadt, Germany).

Fluorimetric measurements were performed with a Fluorolog τ3 spectrofluorometer (Jobin-Yvon/SPEX, Longjumeau, France). Fluorescence spectra were recorded using 390 nm excitation wavelength. The emission values obtained at 540 nm were used for data evaluation. For data collection, photon counting method with 0.1 s integration time was used. Excitation and emission bandwidths were set to 2 nm. To avoid the inner filter effect, a 2 mm thickness of the fluorescent probes with right-angle detection was applied.

Considering the photosensitive character of MTX, the experimental setup was checked to confirm whether the measurement itself induces photodegradation of the MTX. To do that, the MTX samples were illuminated for 2 h using 390 nm light (the excitation wavelength). No considerable spectral changes were obtained which confirm stability of the MTX for the limited time of the measurements [18].

To determine the thermodynamic parameters associated with the complexation reaction of MTX and TAC, samples with constant concentration of MTX (10 µM) and with different concentrations of TAC (0–80 µM) were prepared in DMSO and measured immediately using 390 nm excitation wavelength at temperatures 293.0 K, 296.3 K, 299.7 K, 303.0 K, 306.3 K, 309.7 K and 313.0 K.

### 3.2. Data Evaluation

Stability constants (K, dm^3^/mol) of MTX-TAC complexes were calculated either using the Benesi-Hildebrand equation, assuming 1:1 complex stoichiometry:

(1) $$\frac{I_{0}}{I-I_{0}}=\frac{1}{A}+\frac{1}{A*K*\left[C\right]}\mathrm{lnK}$$

where I_0_ and I are the fluorescence emission intensities of MTX in the absence and in the presence of the host, respectively; [C] is the molar concentration of the host molecule while A is a constant.

Furthermore, association constants of the complex formation were also calculated by nonlinear fitting, based on the fluorescence emission data obtained, employing the HyperQuad2006 program package [19].

To determine the thermodynamic parameters, temperature dependence of the complex stabilities was examined, then the thermodynamic parameters were calculated using the van ’t Hoff equation:

(2) $$\mathrm{lnK}=-\frac{\Delta G}{\mathrm{RT}}=\frac{\Delta H}{\mathrm{RT}}+\frac{\Delta S}{R}$$

where the ΔH and ΔS stand for the enthalpy and entropy changes of the complex formation, respectively, while ΔG is the Gibbs free energy change. R is the gas constant, while T is the temperature in Kelvin.

### 3.3. Modeling Studies

Thermodynamic parameters of the MTX-TAC complexes were determined as follows. The enthalpy change was considered as the energy change calculated by subtracting the total energies of the reactants from the total energies of the products. Similarly, the entropy changes were calculated by subtracting the entropy terms of the reactants from the entropy terms of the products. Calculation of the entropy term is implemented in the HyperChem code [20] as follows: after calculating the vibrational frequencies using the harmonic approximation, the entropy was then determined by the following equation:

(3) $$S_{\mathrm{vib}}=R\underset{i}{\sum }\left\{\frac{h\frac{\nu_{i}}{\mathrm{kT}}}{e^{\left(h\frac{\nu_{i}}{\mathrm{kT}}\right)}-1}\right.\left.-\ln[1-e^{\left(-h\frac{\nu_{i}}{\mathrm{kT}}\right)}]\right\}$$

where the ν_i_ is the frequency of vibration and T is the temperature.

The total energies of the species interacted were calculated at semi-empirical AM1 level using HyperChem 8 code.

## 4. Conclusions

Photoluminescence and molecular modeling studies of inclusion complexes formed by the antimetabolite drug, methotrexate and by the macrocyclic host molecule tetrakis(androst-4-en-3-one-17α-ethinyl)-cavitand highlight two temperature regions where the complexes possess different conformations: opened structure of the host cavitand with free androst-4-en-3-one-17α-ethinyl arms forms enthalpy stabilized structure while the stability of complexes formed by the closed form cavitand is stabilized by the entropy gain. This information can be applicable to design sensitive and selective molecular sensors for methotrexate drug.

## Figures and Tables

**Table 1:** Stability constants determined at different temperatures

Temperature (K)	log K (BH)	log K (HyperQuad)
293.15	4.16	4.13
296.48	4.20	4.18
299.82	4.22	4.20
303.15	4.19	4.20
306.48	3.75	3.72
309.82	3.64	3.61
313.15	2.94	2.92

BH reflects the Benesi-Hildebrand method.

**Table 2:** Thermodynamic parameters of the complex formation between TAC and MTX

Experiments			Modeling		
Temperature (K)	ΔH (kJ mol^−1^)	ΔS (J K^−1^ mol^−1^)	Structure (see Figure 5)	ΔH (kJ mol^−1^)	ΔS (J K^−1^ mol^1^)
293.15–299.82	15.79 ± 0.7	133.56 ± 6	closed	8.43	126.11
303.15–313.15	−210.66 ± 1.7	−614.57 ± 12	opened	−154.42	−214.67

ΔH and ΔS are the enthalpy change and entropy change, respectively.
